# Modeling acid-gas generation from boiling chloride brines

**DOI:** 10.1186/1467-4866-10-11

**Published:** 2009-11-16

**Authors:** Guoxiang Zhang, Nicolas Spycher, Eric Sonnenthal, Carl Steefel

**Affiliations:** 1 Earth Sciences Division at Lawrence Berkeley National Laboratory, MS 90-1116, 1 Cyclotron Road, Berkeley, CA 94720, USA

## Abstract

**Background:**

This study investigates the generation of HCl and other acid gases from boiling calcium chloride dominated waters at atmospheric pressure, primarily using numerical modeling. The main focus of this investigation relates to the long-term geologic disposal of nuclear waste at Yucca Mountain, Nevada, where pore waters around waste-emplacement tunnels are expected to undergo boiling and evaporative concentration as a result of the heat released by spent nuclear fuel. Processes that are modeled include boiling of highly concentrated solutions, gas transport, and gas condensation accompanied by the dissociation of acid gases, causing low-pH condensate.

**Results:**

Simple calculations are first carried out to evaluate condensate pH as a function of HCl gas fugacity and condensed water fraction for a vapor equilibrated with saturated calcium chloride brine at 50-150°C and 1 bar. The distillation of a calcium-chloride-dominated brine is then simulated with a reactive transport model using a brine composition representative of partially evaporated calcium-rich pore waters at Yucca Mountain. Results show a significant increase in boiling temperature from evaporative concentration, as well as low pH in condensates, particularly for dynamic systems where partial condensation takes place, which result in enrichment of HCl in condensates. These results are in qualitative agreement with experimental data from other studies.

**Conclusion:**

The combination of reactive transport with multicomponent brine chemistry to study evaporation, boiling, and the potential for acid gas generation at the proposed Yucca Mountain repository is seen as an improvement relative to previously applied simpler batch evaporation models. This approach allows the evaluation of thermal, hydrological, and chemical (THC) processes in a coupled manner, and modeling of settings much more relevant to actual field conditions than the distillation experiment considered. The actual and modeled distillation experiments do not represent expected conditions in an emplacement drift, but nevertheless illustrate the potential for acid-gas generation at moderate temperatures (<150°C).

## Background

This study was conducted as part of investigations related to the long-term safety assessment of the proposed high-level nuclear waste repository at Yucca Mountain, Nevada. The heat released by the spent nuclear fuel is expected to induce boiling at near-atmospheric pressure of pore water (moisture) present in the unsaturated rock around waste-emplacement tunnels [1-5]. This boiling, as well as the deliquescence of naturally occurring salts in dust, could lead to the formation of residual brines [6,7] at temperatures significantly above the boiling point of pure water [8], possibly accompanied by the release of acid gases such as HCl and formation of acid condensate [9,10]. The potential enhancement of waste package corrosion by the release of HCl and other acid gases from such solutions and subsequent formation of acid condensate was the primary motivation for this study.

Gaseous HCl is often a primary cause of well-casing and pipe-system corrosion in geothermal systems [11-13]. For this reason, many studies have focused on gaseous HCl and acid condensates in geothermal areas. In most instances, the HCl source is volcanic in origin [14-17]. Fournier and Thompson [18] reported the generation of HCl gas through steam-driven hydrolysis of NaCl at 600°C and pressures up to 700 bar, and suggested that the hydrolysis of chloride brines could be at the origin of HCl gas in volcanic and sub-volcanic environments. Bischoff et al. [19], studying the generation of HCl gas in the CaCl_2_-H_2_O system at high temperature (380-500°C and pressure (up to about 800 bar), showed that this system generates significant amounts of HCl in the vapor phase by the hydrolysis of CaCl_2_. These authors concluded this mechanism is likely the source of HCl in steam at the Geysers geothermal system in Northern California.

Very few experiments have been conducted to evaluate the formation of HCl gas in natural systems at atmospheric pressure and (relatively) low temperatures. In the context of geologic disposal of nuclear waste, Pulvirenti et al. [10] distilled a synthetic concentrated CaCl_2_-type water representative of some of the calcium-rich pore waters at Yucca Mountain. These authors observed a number of acid gases released from the concentrated brine, including HCl, Cl_2_, NO, NO_2_, and HNO_3_. The vapor from their distillation experiments produced acid condensate (pH <1). Olsen et al. [9] conducted experiments similar to those of Pulvirenti et al. [10], and also obtained highly acid distillate and brine boiling temperatures exceeding 140°C. In this case, the authors suspected the acid gas produced may have consisted primarily of nitrosyl chloride (NOCl), which would be expected to release HCl upon contact with water [20]. In both these cases, solutions were boiled in closed or partially closed systems that were unlike the conditions in a waste emplacement drift, but nevertheless indicated the generation of acid gases at temperatures significantly lower than is characteristic of geothermal systems.

Understanding the generation of acid gases and condensates is important for protecting metal structures from corrosion in geothermal production systems (and other engineered systems in the vicinity of boiling solutions). Modeling studies can provide insights into these processes and thus can be quite helpful. For example, Helgeson [21] and Glover [22] calculated the theoretical maximum HCl content of a vapor phase released from boiling a deep geothermal brine in an open system. Their results show that the maximum HCl content, as a function of temperature and steam fraction, can reach up to 200 mg/kg. Using a batch model, Andreussi et al. [23] calculated the chemical composition of the first drop of condensate that forms at dew-point temperature through expansion of superheated geothermal steam, with implications for corrosion and scaling in turbines. Their calculation considers the solution boiling-point elevation by referring the measured boiling point of NaCl solutions to an ionic strength that is the same as the solution being studied. More complex modeling studies on acid-gas generation and condensation from boiling chloride brines have not been conducted, at least not in the context of multiphase flow and multicomponent reactive transport, as done here. The objective of the present study is, therefore, to assess these processes as quantitatively as possible under conditions relevant to boiling systems at atmospheric pressures. This is achieved by first presenting simple bounding derivations of condensate pH as a function of vapor loss from a distiller (dynamic system) for a vapor initially at equilibrium with a saturated calcium chloride brine at 50-150°C and 1 bar. A more complex reactive transport model is then applied to simulate the distillation of a calcium-chloride-dominant brine at atmospheric pressure. Results show that acid distillates can be generated from relatively low-temperature gases (< 150°C), with pH values strongly dependent on the amount of water vapor condensing from the gas phase.

## Reactive Transport Model and Thermodynamic Data

The reactive transport simulator TOUGHREACT [[24] and references therein] is used here, extended with a Pitzer activity-coefficient model based on the formulation of Harvie et al. [25] and thermodynamic data from Wolery et al. [26] (as implemented by Alai et al. [7]). For the Ca-Cl system, interactions parameters in this database were derived from [27] (Model 2 of these authors, without separate ion pairs CaCl^+ ^and CaCl_2(aq)_) together with solubility data for CaCl_2 _hydrate salts from [28] and [29]. These data reproduce reasonably well saturation concentrations from [29] for CaCl_2_:2H_2_O up to about 100°C [26], however somewhat underestimate saturations (by 15-20%) at ~150°C. A later revision of this thermodynamic database [30], not available prior to the start of this study, includes data derived from [27] (Model 3 of these authors, with separate ion pairs CaCl^+ ^and CaCl_2(aq)_) that yield more accurate saturations at high temperatures. For HCl(g), reference thermodynamic data are taken from [31] and extrapolated to higher temperatures using SUPCRT92 [32]. The implementation of the Pitzer model in TOUGHREACT is presented in Zhang et al. [33,34], together with comparisons of calculated activity and osmotic coefficients with measured data from the literature [e.g., [35]].

TOUGHREACT computes reactive flow and transport for multicomponent and multiphase systems. In the present case, the multiphase system includes an aqueous phase containing dissolved salts, a gaseous phase (air, water vapor, trace gases), and various solid mineral phases. Therefore, a representative geochemical system is considered, including aqueous speciation, mineral (salts) precipitation and dissolution, and gas generation and dissolution/reaction in condensate. The coupled heating, boiling, vapor phase transport and condensation are modeled in the context of nonisothermal multiphase flow and reactive geochemical transport. The boiling point elevation caused by elevated concentrations of dissolved salts is captured in the model through the lowering of the water-vapor pressure resulting from the decrease in water activity with increasing ionic strength [34]. The numerical model relies on an equation-of-state module that computes the physical properties and phase equilibrium behavior of water and saline solutions, coupled with multiphase flow and transport (advection and diffusion) in the phases involved (liquid or gas). As such, the numerical model simulates "real" evaporation and condensation as a function of temperature, pressure, and system composition, including vapor pressure lowering and PVT effects from condensation and feedback on fluid flow [36].

It should be noted that TOUGHREACT was designed to simulate multiphase flow in porous media (i.e., using Darcy's law), and therefore cannot accurately simulate flow in open conduits (i.e., flow represented with Navier-Stokes equation) such as would be required for the distillation simulation presented below. In this study, flow and transport processes in open conduits are approximated using a high-permeability porous-medium representation. In this respect, simulation results regarding gas flow are regarded as more qualitative than quantitative.

## Preliminary Analysis

Preliminary calculations assuming pure CaCl_2 _solutions were carried out to investigate relationships between salt concentration, HCl gas fugacity (≅ partial pressure), and condensate pH at various temperatures. These calculations are based on analytical derivations that are applied using computed HCl gas fugacities and water activities. The objective is to illustrate, as simply as possible, the effect of partial condensation (vapor loss from a distiller) on condensate pH in a dynamic system. Note that more sophisticated numerical simulations of distillation are presented later, and do not rely on these preliminary analyses.

First, speciation computations were run to determine the pH, HCl gas fugacity, and water activity of solutions saturated with CaCl_2_:2H_2_O (the stable solid phase in the temperature range considered) at 1 bar and temperatures from 50 to 150°C (Table 1). The pH of distillate that would form from the condensation of vapors equilibrated with these solutions was then estimated using simple relationships and assuming ideal behavior, as shown below. The speciation computations were run using the Pitzer model and thermodynamic data mentioned previously. It was noted earlier that above ~100°C, these data yield salt saturations lower than reported values (15-20% at ~150°C). For this reason, results should be viewed as approximate, yielding HCl fugacities and water activities (and consequently condensate pH values) that are rather overestimated than underestimated at elevated temperatures.

**Table 1:** Computed chemical properties of saturated CaCl_2_:2H_2_O solutions at 50-150°C and ~1 bar.

Temperature (°C)	**log f**_**HCl**_ (log bar)	**pH**^**1**^		Water Activity	Ionic Strength (molal)	***P***^**0**^_**sat**_ **(bar)**^**3**^	***P***_**H_2_O**_ **(bar)**^**4**^
50	-5.5	3.0	5.7	0.174	35	0.124	0.022

75	-4.5	2.9	5.3	0.189	36	0.386	0.073

100	-3.7	2.8	4.9	0.200	39	1.014	0.203

125	-3.0	2.8	4.5	0.216	40	2.322	0.502

150	-2.5	2.9	4.2	0.235	42	4.762	1.119

^1 ^pH defined as , with γ being the activity coefficient from the Pitzer model; ^2 ^Also defined as pmH or p[H]; ^3 ^NIST tables [37]; ^4 ^Equation (5)

For a gas mixture including H_2_O and HCl gas, the molar ratio of acid-gas to water is given by(1)

where *P *and *n *stand for pressure and number of moles respectively. Because HCl is highly soluble in water and H_2_O is highly condensable, the ratio *n*_HCl_/*n*_H2O _in the above equation can be assumed to remain essentially the same in the distillate after full (and conservative) condensation. Therefore, we can write(2)

where *m *stands for molality (moles/kg_water_) in the condensate. If the pH of the condensate results only from HCl dissociation, assuming full HCl dissociation and neglecting activity coefficients in the condensate, then(3)

Note, in this case,  and is technically referred to as pmH or p [H]. Combining Equations (1), (2), and (3) then yields(4)

*P*_H2O _can be expressed as a function of the water activity *a*_H2O _and pure water saturation pressure *P*^0^_sat_, thus taking into account vapor-pressure lowering due to salts:(5)

We now consider that only a fraction of H_2_O initially in the gas phase condenses, but that essentially all HCl in the gas phase partitions into the condensed phase (because of the very strong affinity of HCl for water). This would occur in a distiller when steam flows through a condenser with incomplete or poor heat transfer, and HCl is continuously scrubbed from the steam flowing above the condensate. Incomplete condensation can be expressed by multiplying *n*_H2O _by (1-*X*) in Equation (1), where *X *is defined as the (mass) fraction of uncondensed H_2_O (e.g., steam loss from a distiller). Introducing this variable in subsequent equations, and substituting Equation (5) into (4) then yields:(6)

**Figure 1:**
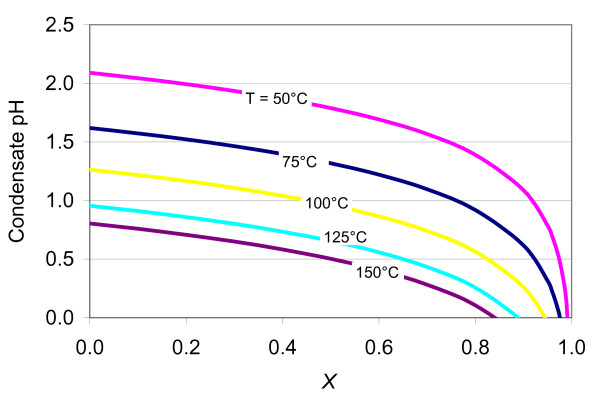
**Calculated pH (technically pmH, Equation 6) of condensates from vapors initially at equilibrium with solutions given in Table 1 (at temperatures shown), as a function of H_2_O vapor mass fraction loss from a distiller (*X*)**. *X *= 0 for full H_2_O condensation; X>0 for partial condensation of a flowing vapor stream (see text).

Equation (6) was used to estimate condensate pH for vapors evolved from the CaCl_2 _solutions shown in Table 1. Results show condensate pH values below 2.5 for all cases, and dropping sharply with elevated steam loss as less water is available to dilute HCl scrubbed from the gas phase (Figure 1).

## Numerical Simulation of Distillation

The distillation of a calcium-chloride-dominant brine was simulated based on an experiment reported by Pulvirenti et al. [10]. These authors distilled at atmospheric pressure a synthetic brine of a composition intended to represent evaporated Yucca Mountain pore water. The actual brine composition was determined by Rosenberg et al. [6] after evaporating a synthetic solution representative of certain types of calcium-rich pore waters at Yucca Mountain and concentrating that solution by a factor of 1,243.

**Figure 2:**
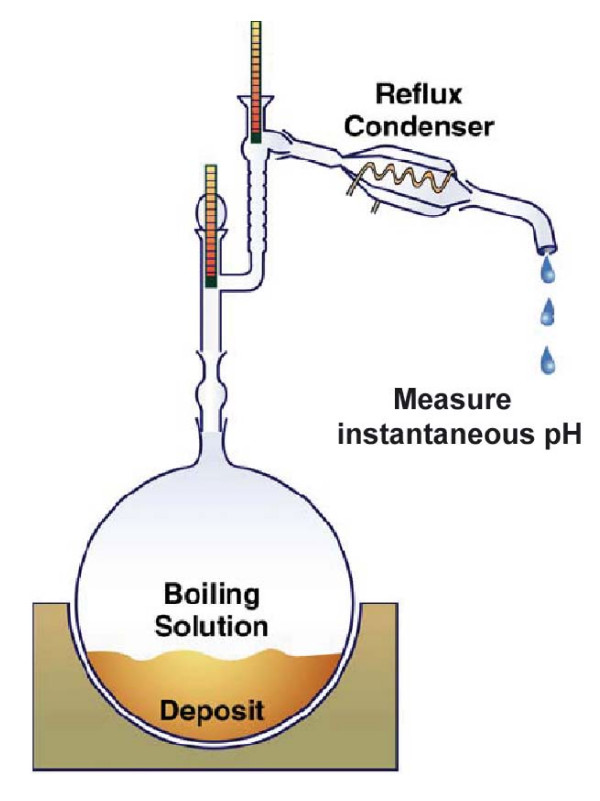
**Schematic illustration of distillation experiment, after Pulvirenti et al**. [10].

Although Pulvirenti et al. [10] give limited detail regarding their distillation experiment, they report sufficient information for us to reasonably reconfigure and simulate an experiment similar to theirs (Figure 2). These authors used an initial volume of 12 L of brine, which was prepared using a mixture of salts (Table 2). The brine was placed in a round-bottom flask on a heating mantle, heated up to 144°C, and distilled until 40 to 250 mL of liquid remained in the flask (corresponding to concentration factors of ~370,000 and ~60,000, respectively, relative to the initial pore water used by Rosenberg et al. [6]). A water-cooled condenser was attached at the top of the flask. The distillate was collected at intervals for (instantaneous) pH measurement. The temperature was also monitored in both the flask and condenser. Towards the end of the distillation experiment, Pulvirenti et al. [10] report that the pH of the condensate droplets fell to 1 and lower. These authors also observed some solids precipitating in the boiling flask, but did not report their composition. Gases were detected using color indicator tubes and included Cl_2_, NO, NO_2_, HNO_3_, and HCl. Analyses of the condensate by Inductively Coupled Plasma Atomic Emission Spectrophotometry (ICP-AES) revealed the presence of large amounts of chloride and nitrate in the condensate.

**Table 2:** Initial brine salt recipe (Pulvirenti et al. [10]).

Solute	Concentration (mg/L)
CaCl_2_:2H_2_O	57767

MgSO_4_:7H_2_O	5382

KNO_3_	4205

NaF	1198

NaHCO_3_	61

SiO_2 _*x*H_2_O (84%SiO_2_)	644

MgCl_2 _6H_2_O	42264

NaCl	14211

KCl	1975

NaOH	120

**Figure 3:**
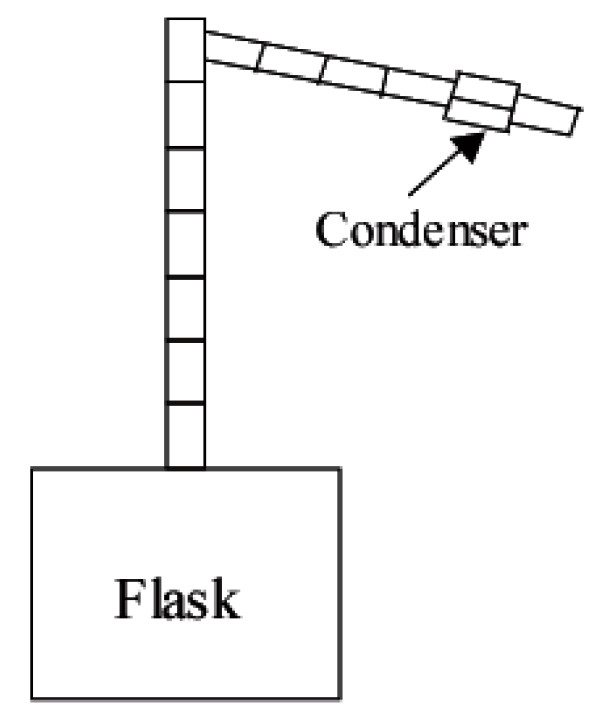
**Discretization of the evaporation/condensation experiment simulated in this study (finite volume model)**. The experimental setup illustrated in Figure 2.

To simulate this experiment, we discretized the distillation setup (Figure 2) using a number of gridblocks (Figure 3). Open spaces (the flask, pipe assembly, and the condenser) were represented by a high-permeability (10^-6 ^m^2^) porous medium without capillarity. The gridblock representing the flask was set with a volume of 15 L, unit porosity, and an initial liquid saturation of 0.8 to yield 12 L of initial solution in the flask. The liquid saturation was set to 0 everywhere else. The gridblocks representing the pipe and the condenser were set with a "porosity" of 0.64 calculated from an arbitrarily chosen pipe outer diameter of 1 cm and inner diameter of 0.8 cm. The diffusion coefficients of gases were calculated as a function of gas molecular weight and diameter, pressure, and temperature [38] with a system tortuosity assumed equal to 1.

The atmosphere was represented using a large gridblock (essentially infinite volume) having constant temperature and pressure (25°C, 1 bar), and connected directly to the gridblock representing the condenser, set at the same conditions. Only the condenser gridblock was allowed contact with the atmosphere (the outlet of the condenser). Initial conditions in the gridblock representing the flask were set at the boiling point of pure water under atmospheric conditions (~100°C, 1 bar). Heat was applied directly to the gridblock representing the flask. No liquid-phase flow was allowed to take place from the flask to the condenser; thus, mass transport resulted only from vapor flow by advection and diffusion. Heat loss by vapor flow out of the system was accounted for. Note that "real" condensation was simulated, meaning that the model relies on a full equation of state coupled to heat flow and fluid flow to predict the amount of condensation taking place by cooling in the condenser. In the present case, most of the generated vapor was predicted to flow into the atmosphere gridblock, with a minor part condensing in the condenser, as discussed later. The distillate was allowed to flow out of the condenser gridblock into the atmosphere gridblock.

The initial brine composition is shown in Table 3. This composition was calculated from the salt recipe in Table 2. The initial brine pH was computed from multicomponent speciation using the known total (stoichiometric) hydrogen ion concentration given by the salt recipe, and assuming an initially atmospheric CO_2 _partial pressure. All dissolved components shown in Table 2 were considered in the simulation. In addition, a number of salts (minerals) were allowed to precipitate in the flask, including calcium chloride (CaCl_2 _and CaCl_2_:2H_2_O), calcite (CaCO_3_), anhydrite (CaSO_4_), niter (KNO_3_), soda niter (NaNO_3_), halite (NaCl), sylvite (KCl), epsomite (MgSO_4_:7H_2_O), Mg(NO_3_)_2_, nahcolite (NaHCO_3_), fluorite (CaF_2_), villiaumite (NaF), carobbite (KF), amorphous silica (SiO_2_) and magnesium chloride (MgCl_2_:4H_2_O). These salts were selected primarily on the basis of the initial salt recipe (Table 2), with added few other common minerals that could form as a result of evaporation. Note that the focus of this study was primarily on the behavior of acid gases in the vapor rather than on the exact prediction of the types and amounts of solids formed in the flask. Gases considered included CO_2_, HCl, HF, and HNO_3_. The initial CO_2 _partial pressure was assumed atmospheric everywhere, and initial partial pressures of HCl, HF, and HNO_3 _gases were set to reflect equilibrium with the solution in the beaker. Away from the beaker, initial partial pressures of acid gases were set to very small, essentially zero values (10^-20 ^bar). Thermodynamic data for these phases, as well as all ion-interaction parameters for dissolved components, were taken from the sources discussed earlier. All reactions were assumed to proceed at equilibrium.

**Table 3:** Initial chemical composition of the synthetic brine used in the numerical simulation, determined from the salt recipe in Table 2.

Components	Concentration (molal^**1**^)
pH	~8 (~100°C)^2^

Ca^+2^	4.2609E-01

Cl^-^	1.5954E+00

F^-^	3.0940E-02

HCO_3_^-^	7.8739E-04

K^+^	7.3828E-02

Mg^+2^	2.4910E-01

Na^+^	2.9866E-01

SO_4_^-2^	2.3678E-02

SiO_2(aq)_	1.3088E-02

NO_3_^-^	4.5102E-02

^1 ^From reported initial salt concentrations in mg/L (Table 2), assuming a solution density ~1.05 kg/L. The number of significant digits is provided only for accurate numerical charge balance and does not reflect the accuracy of the data.

^2 ^Calculated by chemical speciation given input total (stoichiometric) H^+ ^concentration

The time step size was chosen to capture the fast evaporation of the brine (relative to a constant heat input) in the final boiling stages. The simulation was run for a total boiling time of 3.1 days, a point at which the ionic strength of the brine in the flask reached 41 molal and numerical convergence was difficult to achieve. That simulation end point corresponds to a concentration factor of ~54,000 relative to initial pore water.

## Results

**Figure 4:**
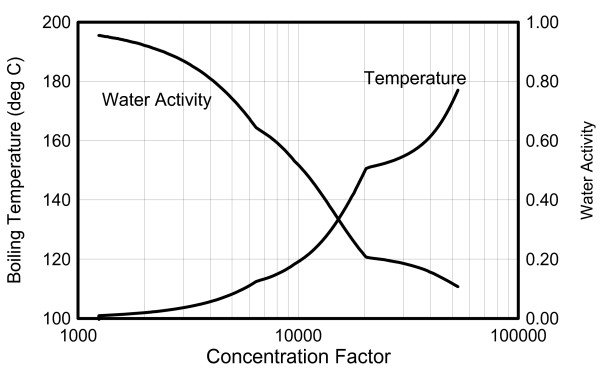
**Simulated boiling temperature and water activity of the brine as a function of concentration factor**.

**Figure 5:**
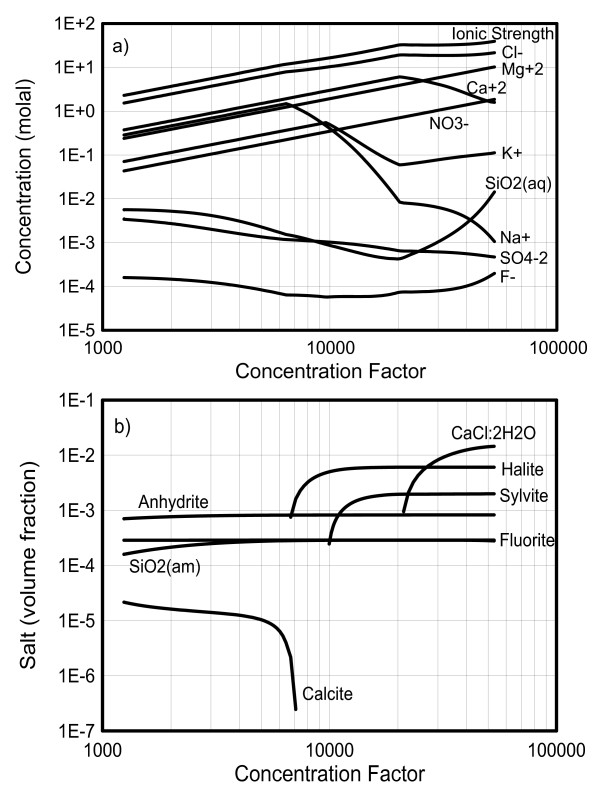
**Simulated concentrations of aqueous components in the boiling brine (a) and predicted salt precipitation (volume fraction change) (b), as a function of concentration factor**.

**Figure 6:**
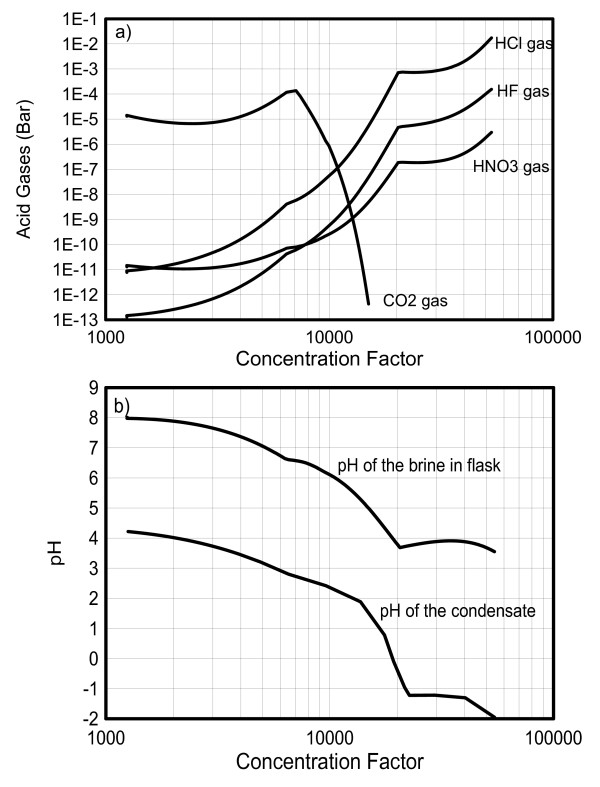
**Simulated acid-gas partial pressures generated from the boiling brine (a), and resulting pH in the brine and (instantaneous) distillate after condensation at 25°C (b), as a function of concentration factor**.

Simulation results are presented below as a function of concentration factor (Figures 4, 5, and 6). The concentration factor can be regarded as the number by which the concentration (molality) of a conservative component in the flask would increase during the distillation process. As mentioned earlier, the starting brine in the simulation has a composition corresponding to a solution already concentrated by a factor of 1,243 relative to the initial pore water (from [6]). For this reason, the concentration factor in all figures is expressed relative to the original pore-water composition (thus starting at 1,243).

The simulated temperature of the brine rises with increasing concentration factor (Figure 4), directly reflecting the effect of vapor-pressure lowering by increasing salt concentrations. Two main breaks in the temperature and water activity trends (Figure 4) reflect the precipitation of halite at a concentration factor around 6,500 (water activity around 0.65) and calcium chloride at a concentration factor of around 21,000 (water activity around 0.2) (Figure 5).

The concentrations of aqueous components increase monotonically before precipitation occurs (Figure 5). After the precipitation of halite, the sodium concentration decreases, but chloride concentration continues to increase, although at a decreased rate. This occurs because the chloride concentration is higher than the sodium concentration in the initial solution (Table 2). Note that the sodium concentration does not remain constant after equilibration with halite because the concentrations of other species still increase with continued boiling, thus affecting activity coefficients and the ionic activity product of halite. The same processes apply to other components. Upon further boiling, the precipitation of calcium chloride removes calcium from the solution, causing the calcium concentration to decrease and chloride concentration to nearly stabilize. Note that sylvite precipitation is predicted to start at a concentration factor close to 9,500, causing an obvious decrease in potassium concentration but essentially producing unnoticeable changes in the temperature and chloride trends. However, at the onset of calcium chloride precipitation, the potassium concentration starts to increase because the precipitation of calcium chloride consumes a lot of choride, causing sylvite to precipitate at a decreased rate. Nitrate salts were not predicted to precipitate in this simulation, which was carried out until numerical convergence was no longer achieved (at concentration factor ~54,000).

The pH of the boiling brine is computed to decrease from an initial value of around 8, with noticeable breaks at pH 6.6 when halite starts to precipitate (concentration factor around 6,500) and pH around 3.7 when calcium chloride forms (concentration factor around 21,000) (Figures 5 and 6). The pH decrease is mostly driven by the evaporative concentration effect on H^+^, as well as the increase in the activity coefficient of H^+ ^at higher ionic strengths, and (to a lesser extent) by the temperature effect on the dissociation of water and bicarbonate. The precipitation of halite and later calcium chloride (the two most dominant salts) are responsible for most of the reduction in the rate of ionic strength increase with evaporation (and thus most of the reduction in the rate of activity coefficient increase and water activity decrease). As a result, the precipitation of these salts coincides with breaks in the pH trend. Note that as a result of decreasing pH, and a more pronounced activity coefficient increase for sulfate (double-charged) than bicarbonate (single-charged), calcite dissolves in favor of calcium sulfate precipitation (e.g., SO_4_^-2 ^+ CaCO_3(s) _+ H^+ ^==> CaSO_4(s) _+ HCO_3_^-^) and eventually totally disappears at a concentration factor of about 7,200.

Three acid gases, HCl, HF, and HNO_3_, are exsolved from the boiling brine in the beaker. Their partial pressure trends as a function of concentration factor (Figure 6) show breaks corresponding to the changes in pH discussed above (Figure 6). These breaks are indirectly induced by halite and calcium chloride precipitation (Figure 5) which also correspond to the temperature breaks observed earlier (Figure 4). The trend of CO_2 _partial pressure is closely related to the decreasing pH in the beaker (Figure 6) and the resulting dissolution of calcite (Figure 5). Initially, the CO_2 _partial pressure drops slightly because more CO_2 _is lost by gas-phase transport than produced by calcite dissolution. However, as the pH decreases further and more calcite dissolves, the CO_2 _partial pressure starts rising. Eventually, when all calcite is consumed, the CO_2 _partial pressure drops sharply as the brine becomes depleted in total dissolved carbonate (the diffusion of atmospheric CO_2 _into the distillation apparatus is not sufficient to maintain atmospheric CO_2 _conditions directly above the beaker).

Condensation occurs in the condenser where the vapor is cooled to 25°C. The acid gases dissolve immediately into the condensate, causing its pH to drop (Figure 6). As discussed earlier, the pH of the condensate is controlled primarily by the fugacities of the acid gases (mostly HCl in this case, Figure 6) and the amount of vapor loss from the distiller. The more vapor loss (with preferential partitioning of HCl into the condensate), the smaller the amounts of condensed water and proportionally higher HCl enrichment in condensate, thus the lower the pH in the condensate (Figure 1). Note that in this simulation, the predicted condensate pH (Figure 6) represents computed values for "instantaneous" condensation (i.e., small amounts of distillate generated at prescribed intervals in time, without allowing continuous accumulation of the distillate). The condensate is allowed to accumulate for a simulated time period around 1.5 minutes. During this time, the amount of vapor flowing out of the condenser is computed to be around 96% of the H_2_O mass flowing into the condenser, yielding very low (negative) condensate pH values at elevated temperatures, in qualitative agreement with the preliminary analyses discussed earlier. When simulations are set up such that the distillate continuously accumulates in the condenser, with more complete condensation of the incoming vapor stream, predicted pH values in the condensate remain above 2.

## Discussion and Conclusion

Model results show low-pH condensates from acid-gas volatilization, as observed in the distillation experiments of Pulvirenti et al. [10]. When it is distilled, the synthetic concentrated brine releases acid gases, in our case predicted to consist primarily of HCl and HF. Two key factors, however, affect the generation of acid condensate: (1) the concentration factor and (2) the amount of condensation taking place, which is a direct function of the amount of vapor loss from the distiller (partial condensation). In our simulations, the volatilization of acid gases in quantities significant to yield condensate pH values below 2 were predicted only at high concentration factors (above >20,000), and only in cases where significant vapor loss (partial condensation) occurred. Note that Alai et al. [7] report no evidence of acid gases in evaporation experiments up to concentration factors ~3,400 using a similar type of water. This is in agreement with our results, which show that at such a lower concentration factor, the predicted partial pressures of these gases are quite low (<10^-12 ^bar for HF, <10^-10 ^for HCl, and <10^-11 ^for HNO_3_).

The two salts most significantly affecting predicted thermal evolution and chemical trends in the brine and vapor are halite and calcium chloride. These salts precipitate in our simulations at concentration factors of ~6,500 and ~21,000, respectively, producing noticeable breaks in the predicted trends of brine pH and temperature, gas partial pressures, and condensate pH (Figures 4, 5, and 6). These breaks occur because the rate of ionic strength increase with evaporation is sharply reduced once these minerals start to precipitate, in turn reducing the rate at which ion activity coefficients increase (and water activity decreases) with increasing evaporation. Upon continuous boiling, the most pronounced break in the trend of increasing temperatures occurs at ~153°C when the brine becomes saturated with respect to calcium chloride. It should be noted that, upon further evaporation, predicted temperatures climbed up to near 180°C, significantly higher than the maximum temperature (~144°C) in the distillation experiment [10]. Because of large uncertainties in ion-interaction parameters for mixtures of multiple salts at elevated temperature, most notably those of the nitrate salts, model results above ~150°C (above concentration factors ~20,000) should be viewed with caution.

The model presented in this study incorporates physical and chemical processes affecting the boiling of saline solutions, including boiling point elevation, salt precipitation, and brine chemistry at elevated ionic strength and temperature. By implementing these processes into an existing reactive transport code, we were able to reproduce, at least semi-quantitatively, the volatilization of acid gases and generation of acid condensate observed in laboratory experiments such as those of Pulvirenti et al. [10] and Olsen et al. [9]. The simulation of vapor flow by advection and diffusion was only approximate because open conduits were approximated by a porous medium with high permeability. Nevertheless, fundamental processes were captured, including evaporative concentration with vapor-pressure lowering, volatilization of HCl, HF, and HNO_3_, and transport followed by dissociation of these gases into vapor condensate.

The combination of reactive transport with multicomponent brine chemistry to study evaporation, boiling, and the potential for acid gas generation at the proposed Yucca Mountain repository is seen as an improvement relative to previously applied simpler batch evaporation models [6,7]. This approach allows the evaluation of thermal, hydrological, and chemical (THC) processes in a coupled manner, and modeling of settings much more relevant to actual field conditions than the distillation experiment considered here. Further modeling work by the authors [34] has integrated the type of brine distillation model presented here into full THC simulations of the near-field within and around waste emplacement tunnels. These simulations show that deleterious effects of acid gases from boiling pore waters at Yucca Mountain are not anticipated because very small amounts of acid gases are generated, that are quickly buffered by the comparatively large rock mass surrounding emplacement tunnels.

## Competing interests

The authors declare that they have no competing interests.

## Authors' contributions

GZ and NS carried out numerical simulations, initial draft, revisions and final compilation of the manuscript. ES and CS helped in numerical simulations and manuscript revision. All authors read and approved the final manuscript.
